# Reconstructing the origin and early evolution of the snake brain

**DOI:** 10.1126/sciadv.adi6888

**Published:** 2023-09-27

**Authors:** Simone Macrì, Ida-Maria Aalto, Rémi Allemand, Nicolas Di-Poï

**Affiliations:** ^1^ Institute of Biotechnology, Helsinki Institute of Life Science, University of Helsinki, 00014 Helsinki, Finland.; ^2^ Department of Anthropology, University of Toronto Scarborough, Toronto, ON M1C 1A4, Canada.

## Abstract

Snakes represent one-eighth of terrestrial vertebrate diversity, encompassing various lifestyles, ecologies, and morphologies. However, the ecological origins and early evolution of snakes are controversial topics in biology. To address the paucity of well-preserved fossils and the caveats of osteological traits for reconstructing snake evolution, we applied a different ecomorphological hypothesis based on high-definition brain reconstructions of extant Squamata. Our predictive models revealed a burrowing lifestyle with opportunistic behavior at the origin of crown snakes, reflecting a complex ancestral mosaic brain pattern. These findings emphasize the importance of quantitatively tracking the phenotypic diversification of soft tissues—including the accurate definition of intact brain morphological traits such as the cerebellum—in understanding snake evolution and vertebrate paleobiology. Furthermore, our study highlights the power of combining extant and extinct species, soft tissue reconstructions, and osteological traits in tracing the deep evolution of not only snakes but also other groups where fossil data are scarce.

## Teaser

The evolution of brain morphological features revealed an opportunistic burrowing behavior at the origin of crown snakes.

## INTRODUCTION

The ecological origin and early evolution of snakes has long been a focus of multiple research fields (ranging from paleontology and phylogenetics to ecomorphology and embryology) and remains one of the most enduring and controversial topics in biology (*1*, *2*). Conflicting ecological hypotheses for stem snakes, including aquatic (*3*–*6*), terrestrial (*7*–*10*), semifossorial (*11*), fossorial (*12*–*15*), or even multiple habitats (*16*, *17*), have been proposed on the basis of cladistic or geometric morphometric analyses of various morphological traits. Central to this debate is the paucity of well-preserved snake fossils (*12*, *13*, *16*, *18*, *19*), the difficulty of deciphering squamate phylogenetics (*20*–*24*), and the rapid diversification rates and astounding anatomical transformations of early snakes related to ecological diversity and complexity (*10*, *17*, *25*–*27*). In other words, attempting to make predictions about the origins of an extremely diversified group poorly represented in the fossil record and accounting for over 12% of terrestrial vertebrate diversity (*28*) (with nearly 4000 extant species) can be extremely challenging.

Because of the nature of fossilization, snake evolution in deep time has originally been documented and assessed using preserved osteological discrete traits in skulls, vertebrae, and limbs (or combinations thereof) rather than the related soft tissues (*12*, *13*, *16*, *18*, *19*). Similarly, recent application of x-ray computed tomography (CT) has yielded quantitative insights into the structure-function relationships of skull and bony inner ear labyrinth elements, thus allowing reconstruction of the evolutionary dynamics and ancestral ecologies (mostly habitats) of snakes from datasets including both extinct and extant species (*10*, *15*, *17*, *29*). Despite recent advances, early snake evolution has remained enigmatic, and different perspectives are essential to illuminate stem snake lineages and infer ancestral ecological behaviors. In particular, because of the fragmentary nature of the oldest snake fossils from the Middle Jurassic–Lower Cretaceous (*16*), analysis of internal soft tissues not necessarily preserved in fossils (*30*) but tightly linked to functional and ecological demands in snakes has the potential to clarify these issues. Tracking brain-behavior relationships through the reconstruction of endocranial cavities (endocasts) in extant and extinct taxa (*31*) has been critical for unraveling major evolutionary transitions in vertebrates (*32*–*34*), including humans (*35*). In contrast, despite the crucial importance of sensory processing and the immense diversity of ecological niches exploited by snakes and squamates in general, little is known about neuroanatomy evolution (*36*–*39*) and brain ecomorphology (*40*–*42*) across this group. Although recent qualitative studies have contributed to our understanding of neurocranium ecomorphology by providing a framework to assess early snake evolution (*38*, *39*), the gap in the evolutionary history of squamates is primarily attributable to the species-dependent and overall low or even absent endocast-brain correspondence in certain squamate brain subdivisions (*40*, *43*) [when compared to encephalized taxa like birds and mammals (*31*)], which thus limits the interpretation of elusive or fossil squamate species (*38*, *39*, *42*). Here, we assessed brain phenotypic diversification in the context of snake origins by quantitatively tracking key morphological features of soft tissues in intact whole-brain models of extant squamate species and by re-evaluating the few available endocasts of fossil stem snakes (*11*, *19*).

## RESULTS

### Squamate brain complexity and locomotion

**Fig. 1. F1:**
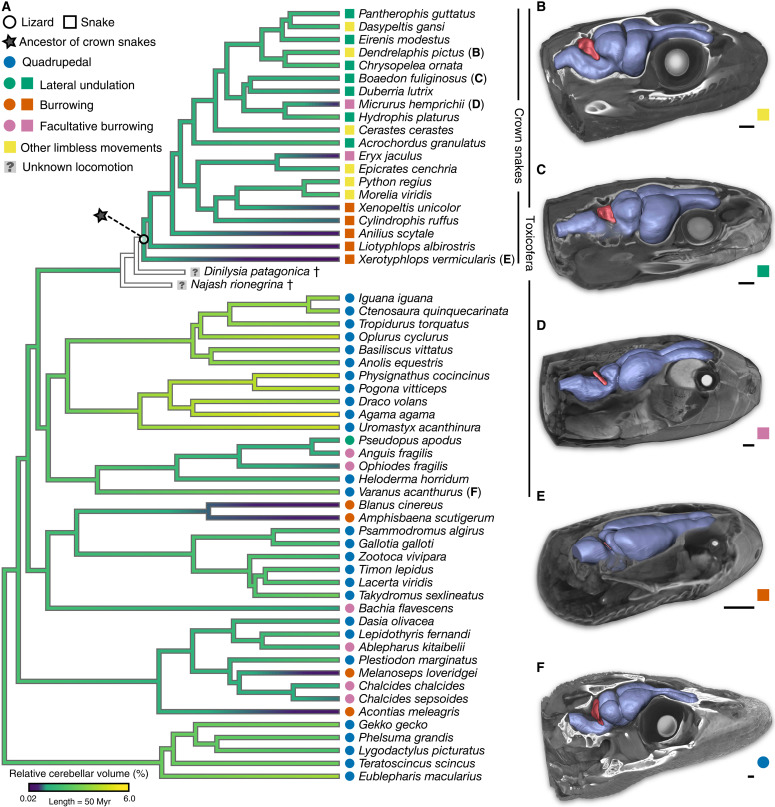
Squamate phylogeny and brain diversity. (**A**) Time-calibrated phylogeny of all extant snake and lizard species used in morphometric and volumetric analyses, derived from the most inclusive phylogenetic study on squamates (*44*). Relative cerebellar volume expressed as cerebellum–to–whole brain percentage is mapped on the tree. The two relatively well-preserved fossil taxa (*D. patagonica* and *N. rionegrina*) included in this study could not be used in phylogenetic analyses of morphological datasets because of the limited information about the cerebellar region, and their phylogenetic position is shown on the basis of recent morphological and molecular evidence on early squamate evolution (*24*). Toxicofera lineages (Serpentes, Anguimorpha, and Iguania) closely related to the origin of modern crown snakes are indicated. The five major locomotor modes for snakes (colored squares) and/or lizards (colored circles), as defined on the basis of anatomical features, habitat use, energy efficiency, and movement type, are represented by the same color code throughout the entire manuscript; similarly, the same symbol (black star) indicates the position of the internal node corresponding to the hypothetical ancestor of crown snakes. (**B** to **F**) High-definition 3D whole-brain models in iodine-stained adult heads highlighting the brain morphology and cerebellum structure (red color) of selected representative Toxicofera at the indicated position in the phylogenetic tree: *Dendrelaphis pictus* (B), *Boaedon fuliginosus* (C), *Micrurus hemprichii* (D), *Xerotyphlops vermicularis* (E), and *Varanus acanthurus* (F). Please note the small and barely detectable cerebellum of the *X. vermicularis*. Scale bars, 1 mm (B to F). Myr, million years.

To assess the ecomorphological diversification of the snake brain, we used high-definition three-dimensional (3D) whole-brain models based on our previously published contrast-enhanced x-ray CT and manual brain segmentation protocol (*41*), which allows accurate preservation of soft tissue integrity and reliable reproduction and quantification of encephalic morphological traits. We generated the most comprehensive dataset of squamate brain reconstructions [58 extant species; approximately 50% increase compared to our previous study (*41*)] by including 3D imaging data from basal snakes and other major Toxicofera lineages (Anguimorpha and Iguania) closely related to the origin of modern snakes (*44*) (>60% of our dataset; Fig. 1A and table S1). Furthermore, instead of the historical and highly debated ancestral habitat hypothesis, we rather exploited the strong correlations between a well-defined ecological behavior—locomotion—and brain morphological features (*41*) as an alternative ecomorphological perspective to assist in clarifying the ecological origins of snakes (Fig. 1A). We expected that classification of major squamate locomotor patterns based on a combination of well-documented cranial and postcranial anatomical characters, energy efficiency features, habitat preference, and movement type (table S1) could provide a comprehensive understanding of early snake evolution. For instance, in addition to quadrupedal gait, a global inspection of our 3D models confirmed the existence of four types of limbless or limb-reduced locomotor patterns—burrowing, facultative burrowing, lateral undulation, and other limbless movements—associated with noticeable changes in the morphology, volume, and spatial configuration of squamate brain subdivisions (Fig. 1, B to F).

**Fig. 2.**
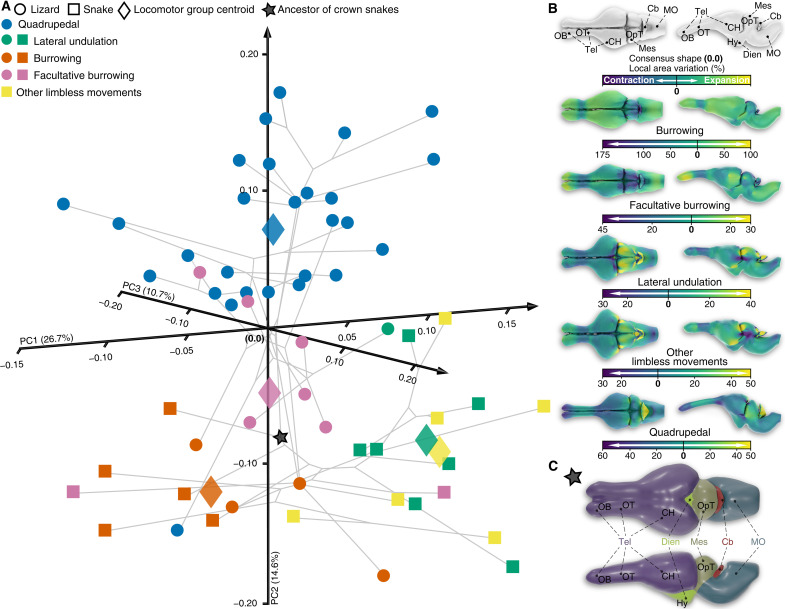
Origin and diversification of whole-brain shape in squamates. (**A**) 3D phylomorphospace representing whole-brain shape distribution of extant snakes (colored squares) and lizards (colored circles) with different locomotor modes (see color code and symbols in top left corner). Numbers in parentheses indicate the percentage of variance explained by each of the main PC axes, and centroids are shown for each locomotor group (colored rhombus). The estimated position of the ancestor of crown snakes is marked by a black star. (**B**) Warped whole-brain surfaces, representing the centroid shape configuration for each indicated locomotor behavior, are shown in dorsal (left) and lateral (right) views. Color gradient, ranging from blue to yellow, reflects the local area changes (in percentages) from the overall consensus shape. (**C**) Warped whole-brain surface corresponding to the reconstructed ancestor of crown snakes is shown in dorsal (top) and lateral views (bottom). Major brain subdivisions are highlighted in different colors. Anatomical abbreviations: Cb, cerebellum; CH, cerebral hemisphere; Dien, diencephalon; Hy, hypothalamic/infundibular region; Mes, mesencephalon; MO, medulla oblongata; OB, olfactory bulb; OpT, optic tectum; OT, olfactory tract; Tel, telencephalon.

Our landmark-based principal component (PC) analysis of whole-brain shape in a phylogenetic context confirms differences among locomotor categories, despite the existence of some overlaps between certain groups. Notably, a gradual morphological transition occurs within limbless or limb-reduced squamates along the PC1 axis, from species adopting burrowing locomotor modalities to those performing surface locomotion (including the overlap of lateral undulation and other limbless movements), passing through intermediate forms exhibited by facultative burrowers (Fig. 2A and fig. S2). Instead, PC2 distinguishes limbless or limb-reduced locomotor behaviors from quadrupedal locomotion (Fig. 2A). The reconstructed whole-brain mean shapes relative to each locomotor mode highlight group-specific brain configurations, as visualized by local area deviations from the entire dataset consensus shape (Fig. 2B). Burrowing snakes and lizards exhibit rostro-caudally elongated cerebral hemispheres that are connected to relatively large olfactory bulbs via short olfactory tracts. Their optic tectum is laterally compressed, with its lateral width largely exceeded by that of a compact medulla oblongata, while their diencephalon is characterized by both a limited dorso-ventral extension and a flattened hypothalamic/infundibular region. It is worth noting that similar expansions or compressions in corresponding regions have been observed in CT-based endocasts of fossorial snake species (*38*–*40*). Furthermore, the cerebellum of burrowing species is extremely reduced in size, nearly triangular, and does not show any noticeable curvature along either the pial or ventricular surface. Compared to lizards and snakes fully adapted to burrowing, facultative burrowing species exhibit relatively larger tectal hemispheres, elongated olfactory tracts and bulbs, and a wider cerebellum with concave pial surface. These observations reveal a contrast with the overall similarities observed in the endocasts of fossorial and cryptozoic forms. This contrast potentially confirms the presence of features in certain brain regions associated with the midbrain and hindbrain that are masked or barely recognizable in endocasts (*38*). Lateral undulating species and snakes adopting other modes of limbless locomotion exhibit substantial similarities in the morphological configuration of their brains. They share rostro-caudally compressed but dorso-ventrally and laterally expanded cerebral hemispheres, straight and thick olfactory tracts, large olfactory bulbs, and a well-developed optic tectum along the antero-posterior axis. These morphologies include several features that were previously recognized as a “surface” cerebrotype based on endocasts (*38*). The cerebellum, trapezoidal in shape, extends almost parallel to the medulla oblongata, rather than perpendicular as in other locomotor groups. Last, quadrupedal lizards exhibit long, curved, and tapering olfactory tracts, antero-posteriorly compressed and laterally protruding tectal hemispheres, and a well-developed, shell-shaped cerebellum.

### Mosaic brains at the origin of crown snakes

When compared to the consensus shape, the cerebellum shows the largest variation in local area (Fig. 2B). This variation ranges from a 175% contraction in burrowing species to a 50% expansion in quadrupedal lizards, confirming the tight link between some brain regions and locomotion in squamates. Consistent with this pattern, and although significant effects of both allometry (percentage shape predicted = 7.9%, *P* value = 0.0015) and phylogeny (*K* value = 0.72, *P* value = 0.001) were identified (table S2), a significant influence of locomotion on whole-brain shape was obtained using allometry-corrected phylogenetic analysis of variance (ANOVA; table S3). However, post hoc pairwise comparisons only reveal significant morphological differences between a few locomotor modes (table S4), including between burrowing or facultative burrowing and other limbless movements. This suggests that whole-brain shape parameters may be a weak indicator of squamate locomotion, at least based on the squamate dataset used in this study. As expected, we could not recover with high confidence the locomotor behavior of the hypothetical ancestor of crown snakes using both linear discriminant analysis (LDA) and typicality probability based on the Mahalanobis distance (tables S5 and S6).

The inconclusive predictor ability of whole-brain shape data and the position of the hypothetical crown-snake ancestor in phylomorphospace—intermediate between burrowing and facultative burrowing forms—are intriguing. Although they may be linked to an incomplete sampling of key snake taxa, they may also derive from the complex relationship between neuroanatomical structures and their function, with morphological changes likely reflecting either multilocomotion abilities or mosaic brain changes in response to various ecological factors or behaviors, extending beyond locomotion. In support of this, various correlations exist between the shape of individual squamate brain subdivisions and locomotor modes, with the cerebellum and diencephalon exhibiting the most highly significant values (*P* values < 0.005; table S3). More informatively, our warping-based reconstruction of the ancestral snake brain highlights morphological features common to all limbless burrowing species [also known as “burrower” cerebrotype (*41*)], including a thin, flat, nearly triangular and small cerebellum, a poorly developed optic tectum, and a compact and wide medulla oblongata. However, other traits like the well-defined, thick, and straight olfactory tracts, the ventrally protruding diencephalon, and especially the rostro-caudally compressed and laterally expanded cerebral hemispheres contrast with an underground lifestyle (Fig. 2C). These observations potentially indicate an opportunistic lifestyle at the origin of crown snakes, with a reconstructed crown snake ancestor fully adapted for burrowing (based on structures such as the cerebellum shape) but not exclusively living underground (as suggested by the data on whole-brain and other brain subdivisions). A strictly burrowing lifestyle would be expected to have a notable impact on various brain regions associated with sensory processing and integration, by reducing the significance of visual stimuli and instead increase reliance on other sensory modalities like audition, touch, chemosensation, and olfaction. The opportunistic hypothesis is further supported by the lack of influence of locomotion on the shape of the mesencephalon (table S3), which plays a crucial role in sensory processing by integrating sensory information from various sources.

### Cerebellum: Key predictor of locomotion

**Fig. 3.**
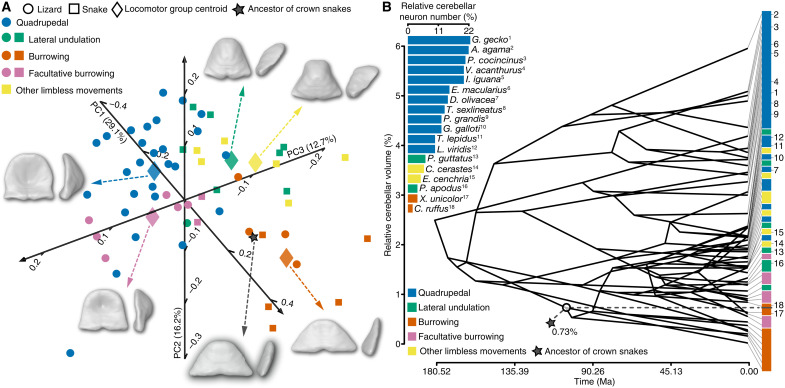
Origin and diversification of cerebellar morphology and cellular content in squamates. (**A**) 3D plot of PC scores showing the shape distribution of isolated cerebella from snakes (colored squares) and lizards (colored circles) with different locomotor behaviors (see color code and symbols in top left corner). Numbers in parentheses indicate the percentage of variance explained by each of the PC axes. Warped surfaces of cerebella representing the centroid shape configuration for each locomotor behavior (colored rhombus) or the reconstructed ancestor of crown snakes (black star) are shown in pial (left) and lateral views (right). (**B**) Variation in the cerebellum–to–whole brain volume ratio (in percentages) for extant species was mapped onto phylogeny presented in Fig. 1, with ancestral state estimates at each internal node. The time axis is expressed in units of millions of years ago. A magnified view of the relative cerebellar volume range for each locomotor mode (see color code in bottom left corner) and the exact position of the reconstructed ancestor of crown snakes (0.73%; black star and dashed line) are shown on the right part of the plot. Numbers are positioned in correspondence to the cerebellar volume ratio values relative to the 18 species with similar or different locomotor modes, which have been further characterized by cerebellar neuron density (expressed as cerebellum–to–whole brain percentage, see top left inset graph). Note the decreased relative cerebellar volumes in all nonquadrupedal limbless snake and lizard species.

To further clarify the locomotor mode of the hypothetical ancestor of crown snakes, we refined our quantitative analysis by combining fixed landmarks and sliding semilandmarks on isolated 3D cerebellar models, thus allowing detailed shape sampling (fig. S1). The morphology of the cerebellum, together with other cerebellar neuroanatomical features (ranging from relative size to fine cellular architecture) has, in fact, been shown to represent a powerful predictor of squamate locomotor behavior because of its critical role in sensory-motor control (*41*). As expected, the refined sampling increased the significant influence of locomotion on cerebellar shape (table S3). Notably, our data indicated morphological differences among various ecological categories, including between burrowing and all other locomotor modes (table S4). This trend was also reflected in the cerebellum phylomorphospace, where limbless burrowing species conspicuously segregate from other locomotor groups, clustering near the positive end of the PC1 axis (Fig. 3A). In contrast, all other locomotor groups partially overlapped in different quadrants of the phylomorphospace (Fig. 3A and fig. S3).

As anticipated from the reconstruction of the whole-brain pattern (see Fig. 2C), the position of the hypothetical crown-snake ancestor in the cerebellum phylomorphospace fell within the cluster of burrowing species (Fig. 3A). Ancestral snake locomotor behavior was predicted with high confidence as burrowing (>93 and >96% of likelihood with LDA and typicality probability test, respectively; tables S5 and S6). Consistent with this, the reconstructed ancestral snake cerebellum exhibited all hallmarks featured by the cerebellum of burrowing species, including a thin, triangular, and flat morphology (Fig. 3A). As another strong indicator of locomotor behavior (*41*), our analysis of volumetric data indicated a substantial divergence in cerebellar size both in absolute and relative terms, with burrowing species showing a significantly reduced cerebellum–to–whole brain volume ratio based on phylogenetic ANOVA (table S7). Using a maximum-likelihood estimator assuming the tree as unit branch lengths, the relative cerebellar volume of the ancestral snake was predicted within the range of burrowing species, in agreement with morphological data (Fig. 3B). However, the estimated relative cerebellar size was relatively higher than in strictly fossorial species such as scolecophidian snakes and amphisbaenian lizards, which have likely undergone further miniaturization (*45*, *46*) including in the cerebellar region (Fig. 1 and table S1). In addition, the conflicting results obtained from LDA and typicality probability analysis on the ancestral cerebellar volume, with predictions of burrowing or facultative burrowing locomotor behavior, respectively (tables S8 and S9), further suggest a nonexclusive underground lifestyle at the origin of crown snakes. Although not statistically assessed here because of the relatively small number of species in some ecological categories, the distribution of additional cerebellar features, including relative neuron content and neuron density, appears to correlate with not only cerebellar size (*47*) but also locomotor modes. In particular, the decrease in relative cerebellar volume was concomitant with a severe reduction in cerebellum–to–whole brain neuron percentage in limbless or limb-reduced lizards and snakes, as exemplified by burrowing species positioning at extreme positions (Figs. 1 and 3B). On the basis of the cerebellar size of the snake ancestor, estimated within the volume range of the two burrowing snakes *Xenopeltis unicolor* and *Cylindrophis ruffus*, these cellular data suggest a relatively low proportion of cerebellar neurons at the origin of crown snakes (<3.5% of brain neurons) when compared with quadrupedal lizards (ranging from 10 to 33%). Similarly, although brain volumetric data do not necessarily correlate with body size, the estimated relative cerebellar volume for the snake ancestor would indicate a body size comparable to basal alethinophidian snakes adapted for burrowing [80 to 130 cm based on extensive lepidosaur data (*48*)], in agreement with not only the reconstructed size of early snake skulls (*17*) but also the range of skeletal fragments recovered for Cretaceous fossorial snakes such as *Coniophis precedens* (*13*).

### Limitations of fossil snake endocasts

**Fig. 4.**
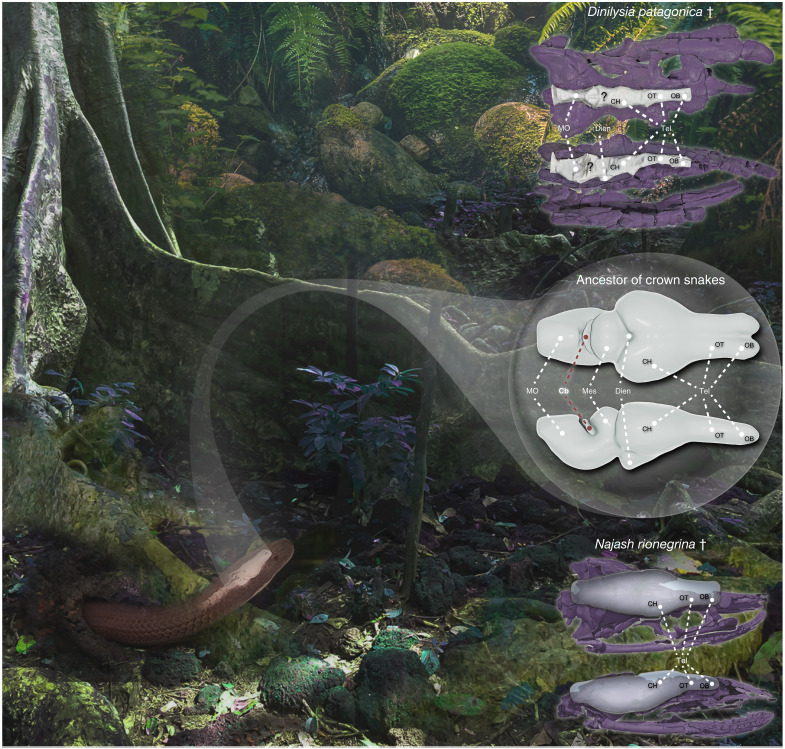
Lifestyle and brain reconstructions of the hypothetical ancestor of crown snakes. Artistic representation of the opportunistic burrowing behavior of the hypothetical ancestor of crown snakes, as predicted from reconstructed high-definition 3D whole-brain models (highlighted middle right) shown in dorsal (top) and lateral (bottom) views. In comparison, the reconstruction of the first natural endocast of a fossil snake (*D. patagonica*, top right) displays a divergent morphological configuration of the telencephalon (Tel) and provides no or limited information about the diencephalon (Dien), mesencephalon (Mes), cerebellum (Cb), and medulla oblongata (MO) regions. In contrast, the estimated endocast of *N. rionegrina* (bottom right) shows similarities with the hypothetical ancestor of crown snakes in several distinguishable telencephalic regions, including cerebral hemisphere (CH), olfactory bulb (OB), and olfactory tract (OT). Reconstructions of the fossil skulls (purple color) are shown around the cranial endocasts. Snake ancestor body segment posterior to rendered vertebrae photo credit: Neptalí Ramírez Marcial, iNaturalist (https://www.inaturalist.org/photos/80198231) (adapted from original, CC BY 4.0 https://creativecommons.org/licenses/by/4.0/).

Although the Upper Cretaceous snakes *Dinilysia patagonica* and *Najash rionegrina* may not represent the ancestral snake condition (*16*), the numerous cranial remains uncovered for these two species are central to predicting the early evolution of modern snakes (*11*, *12*, *17*–*20*) (see phylogenetic positions in Fig. 1). However, even the analyses of an exceptionally well-preserved endocast of *D. patagonica*, including both bony labyrinth and braincase regions, have indicated conflicting or unpredictable ancestral habitat preferences for stem snakes (*11*, *15*, *29*). Here, we show that quantitative assessment of cerebellar morphology has strong potential to clarify the ecological origin of modern snakes. Yet, our endocast reconstruction of *D. patagonica* (*11*) provided limited or no information about the morphology or the transition boundary in the midbrain and hindbrain regions (Fig. 4 and fig. S4). As expected from the situation in extant snakes (*38*–*40*, *43*) and recently observed in CT-based renderings of multiple *D. patagonica* specimens (*38*), only the olfactory tracts and bulbs, the cerebral hemispheres, and some morphological traits of the medulla oblongata and midbrain roof are distinguishable in preserved fossil endocasts. Consequently, any direct assessment of cerebellar ecomorphology in extinct species or even the integration of geometric morphometric data from endocasts with intact (or partial) whole brains is technically challenging as form and shape variations may differ or be exaggerated. Notably, the identifiable brain regions of *D. patagonica* such as the extremely long and curved olfactory tracts, the elongated olfactory bulbs, and the laterally compressed cerebral hemispheres are markedly different from our ancestral reconstruction and represent, in fact, an unusual combination for snakes (*38*). To some extent, the overall elongated endocast morphology combined with both poorly developed cerebral hemispheres along the medio-lateral axis and a noticeable pituitary could be more reminiscent of features found in some surface-dwelling (*38*) or marine snake species (*40*). These observations are consistent with the estimated body length (*38*) and the inferred dual terrestrial and aquatic adaptations based on inner ear morphology (*29*) in *D. patagonica*. However, the estimated gross configuration of the *N. rionegrina* forebrain, including straight and thick olfactory tracts and compact and laterally expanded cerebral hemispheres, resembles that of our reconstructed snake ancestor (Fig. 4). These analogies support similar ancestral morphological and functional adaptations to a burrowing or opportunistic lifestyle, as predicted on the basis of cranial and vertebral morphological traits in *N. rionegrina* (*12*). However, they contrast with the recently described unusual gross anatomy of the brain, similar to that observed *D. patagonica*, found in this snake (*38*). Specifically, the cerebral hemispheres and their width/length ratio exhibit notable differences between the two fossils.

## DISCUSSION

Our broad-scale studies integrating ecological, morphological, volumetric, cellular, phylogenetic, and paleontological data provide a comprehensive view of early snake brain organization and evolutionary specializations from an unexplored perspective. Specifically, using precise cerebellar shape, relative volume, and neuronal content as complementary predictors of locomotor behaviors, we revealed that the hypothetical ancestor of modern snakes was fully adapted for burrowing. Although other predictive cranial osteological models favor a burrowing or fossorial origin of modern snakes (*15*, *17*), our study refutes a strictly underground lifestyle. The inconclusive predictor ability of whole-brain shape data, the estimated ancestral morphology of certain brain subdivisions, and the inferred ancestral cerebellar size all indicate an opportunistic behavior beyond the predicted burrowing capacity. Such a mosaic brain pattern in the crown-snake ancestor may reflect the impact of multiple behavioral or ecological factors, including variation of food habits, exploitation of surface macrohabitats, and some degree of foraging above ground (or combinations thereof), as observed in many basal alethinophidian snakes (*49*) and in some Cretaceous stem snakes such as *N. rionegrina* (*12*, *13*). Such a specialized burrowing locomotion pattern with a predicted opportunistic behavior corroborates recent hypotheses that early snake evolution has involved episodes of occupation of diverse environments (*16*, *17*, *25*, *29*), which also highlights the importance of distinct methodological and ecological perspectives for unraveling the origin of snakes. As exemplified by the predicted ancestral mosaic brain pattern and the failure of well-preserved *D. patagonica* endocast to represent the morphology of midbrain and hindbrain regions, we revealed the power of combining extant and extinct species, soft tissue reconstructions, and osteological traits in tracing brain evolution, ecomorphology, and the ecological origins of snakes from a neurobiological perspective. Furthermore, our study provides an alternative holistic framework that can be reinforced by the inclusion of additional key basal fossorial alethinophidian extant taxa, such as tropidophiids and uropeltids (*38*), as well as future paleontological discoveries, including fossilized brains (*30*). Last, analysis of not only external but also internal brain features, including neuronal parameters such as morphology, spatial arrangement, and connectivity, can also improve the robustness of our framework.

## MATERIALS AND METHODS

### Data collection and generation of 3D whole-brain models

Iodine-based contrast-enhanced x-ray CT scan of adult heads were obtained from 58 extant squamate species (38 lizards and 20 snakes). Specimens were primarily sampled from our previous work (*41*) and from specialized retailers, collections at the Finnish Museum of Natural History (Finland), and MorphoSource data repository (www.morphosource.org) (table S1). Fixation and iodine staining of fresh and museum samples were performed as before (*41*). Only well-preserved soft tissues with no artifacts such as deformation and shrinkage were used. Different samples per species have been used whenever possible to confirm both the fixation/staining procedures and brain reconstructions. We referred to the December 2022 version of the Reptile Database (www.reptile-database.org) for taxonomical reference and squamate species numbers. The CT data of the most complete braincase of the fossil *D. patagonica* (specimen MACN-RN 1013) were downloaded from MorphoSource and used to reconstruct the 3D endocast. The *N. rionegrina* endocast was estimated on the basis of 2D views and 3D skull surface model of specimen MPCA 500 (*19*), providing a good approximation of the configuration of the telencephalon (olfactory bulbs, olfactory tracts, and cerebral hemispheres). As the salient morphological features of midbrain, dorsal diencephalon, and cerebellum are almost completely indistinguishable in squamate endocasts, it is likely that even the use of raw fossil materials may not yield an accurate representation of these brain regions in *N. rionegrina*. Extant species were carefully selected to cover all major groups of squamates (*44*), including basal snakes and Toxicofera species, thus allowing a comprehensive representation of the diversity of locomotor behaviors (table S1). All reptile captive breedings and experiments were done following international standards and were approved by the Laboratory Animal Centre of the University of Helsinki and/or the National Animal Experiment Board in Finland (license numbers KEK21-004, ESAVI/7484/04.10.07/2016, and ESAVI/5416/2021).

Categorization of locomotor groups was performed as defined before (*41*) by cross-correlating data from cranial and postcranial anatomical features (skull shape, limb reduction, and body elongation), habitat modes (aquatic, terrestrial, burrower, facultative burrower, arboreal, semiarboreal, and aerial), movement types associated with locomotor performance (lateral undulation, rectilinear, sidewinding, concertina, modified concertina, and quadrupedal), and morphological features associated with energy efficiency (tail size, neck constriction, smoothness, or microornamentation of scales). In the context of snake origins, five major locomotor groups were defined on the basis of these criteria, including a quadrupedal category (arboreal, semiarboreal, or terrestrial species using quadrupedal locomotion) used as outgroup and a limbless or limb-reduced category further divided into the following four subgroups: burrowing (species living and foraging primarily underground and using modified concertina or rectilinear locomotion), facultative burrowing (terrestrial species with some leaf-litter or sand-swimming behavior using slow lateral undulation locomotion), lateral undulation (terrestrial, aquatic, or arboreal species using lateral undulation locomotion), and other limbless movements (terrestrial or arboreal species using rectilinear, arboreal concertina, or sidewinding locomotion). 3D volume rendering and manual segmentation of brain subdivisions according to anatomical and spatial relationships were performed using the software Amira 5.5.0 (Thermo Fisher Scientific, USA) as described before (*41*). Most brain structures have been included in the reconstructions, except for the poorly discernible pituitary and pineal glands. Whole brain and cerebellum were individually segmented in all specimens, allowing both volumetric and morphometric quantification of these structures.

### Landmarking and high-density sampling of cerebellar shape

Seventy-three fixed anatomical landmarks [61 derived from our previous study (*41*); fig. S1], subdivided in discrete clusters defining the 3D profile of the five major brain subdivisions (telencephalon, diencephalon, mesencephalon, cerebellum, and medulla oblongata), were used to characterize the shape of the squamate whole brain and its individual partitions. Parallel to the morphological characterization of the cerebellum on the whole brain (15 fixed landmarks; fig. S1[Fig F1]), we exploited the isolated cerebellum models to perform a detailed and accurate representation of cerebellar morphological traits. For this purpose, high-density geometric morphometrics (*10*, *50*, *51*) was used by first adding five fixed landmarks at the mesencephalon-cerebellum junction, as defined in our previous study (*41*) (fig. S1[Fig F1]), and then by applying 18 curves (each composed of 20 equidistant semilandmarks and anchored between two consecutive fixed landmarks along the cerebellar edge) on each reconstructed squamate cerebellum. In addition, two distinct surface patches consisting of 56 and 57 regularly spaced semilandmarks were created for the pial and ventricular surface, respectively (fig. S1[Fig F1]). Such a configuration resulted in a total of 493 landmarks on each isolated cerebellum model (20 fixed landmarks, 360 curve semilandmarks, and 113 surface semilandmarks). In contrast to fixed landmarks and curves, surface semilandmark patches were digitized on a single template specimen (*Bachia flavescens*) and then semiautomatically projected onto all other 3D meshes. To maximize surface semilandmark consistency across species, the two patches were independently projected on the corresponding cerebellar surface of all samples in our dataset. Following patch projection, all semilandmarks were slid to minimize bending energy (*52*, *53*). Curve and surface semilandmarks were generated using the extension Slicermorph (*54*) in the platform 3D Slicer v5.0.2 (*55*). Template creation, surface semilandmark patch projection, and semilandmark sliding were implemented in the package Morpho v2.1.0 (*56*) with R (*57*).

### Geometric morphometrics and statistical analyses

To assess whole-brain and cerebellum shape evolution, a generalized Procrustes analysis (GPA) was conducted on landmark configurations, and a phylomorphospace was generated by plotting the main PC scores on the most recent and inclusive phylogenetic tree for extant squamates (*44*). The two relatively well-preserved fossil taxa (*D. patagonica* and *N. rionegrina*) included in this study could not be used in phylogenetic analyses of morphological datasets because of the limited information about the cerebellar region. The ancestral shapes of the internal nodes were estimated using weighted squared-change parsimony for a Brownian motion model of evolution, where transition costs were weighted by the inverse edge lengths, equivalent to the maximum-likelihood estimator (*58*, *59*). Phylogenetic signal was calculated using a multivariate generalized *K*-statistic (*60*). To correct for significant allometric effects, statistical analyses were performed on residuals of the multivariate regression of shape onto size, and phylogenetic relatedness was corrected in analyses requiring independent observations across different species. The influence of locomotor modes on brain shape was assessed with post hoc pairwise tests, using phylogenetic ANOVA based on generalized least squares approach (*61*). GPA and subsequent analyses were performed in the R package geomorph v4.0.5 (*62*). Ancestral snake ecologies were predicted from phylogeny/allometry-corrected shape and volume parameters, implementing both LDA in the R packages MASS v7.3-54 (*63*) and caret v6.0-93 (*64*) and typicality probability test (*65*, *66*) in the package Morpho v2.1.0 (*56*). The number of statistically significant PCs, selected to infer ancestral snake locomotor behavior in predictive tests, was determined according to the broken stick model (*67*) in the R package PCDimension v1.1.13 (*68*).

### Locomotor group mean shapes and ancestral whole-brain and cerebellum reconstructions

To reconstruct the mean shape configuration of the whole brain and cerebellum for each locomotor group, the closest specimen to the overall mean shape in multidimensional space was first identified via the function “findMeanSpec” in geomorph package (*Chalcides chalcides* and *Iguana iguana* for whole brain and cerebellum, respectively), and a 3D brain model of this specimen was warped to the consensus (0.0). The obtained overall mean shape was subsequently deformed toward the centroid of each locomotor category to retrieve locomotor group–specific mean shapes. In a similar way, the 3D surfaces of the specimens more closely resembling the estimated whole-brain and cerebellar morphologies of the hypothetical snake ancestor (*Anilius scytale* and *X. unicolor* for whole brain and cerebellum, respectively) were warped toward the snake ancestor node in the multidimensional phylomorphospace, thus allowing a reconstruction of the ancestral snake brain and cerebellum. 3D model deformations, based on thin-plate spline method (*69*), were implemented in the R package Morpho v2.1.0 (*56*). Morphological deviations from the whole-brain consensus were mapped onto each locomotor group centroid shape as variations (expansion or contraction) of local mesh area, using the R packages Arothron v2.0.3 (*70*) and viridis v.0.6.2 (*71*).

### Volumetric and cellular quantification

Volumetric quantification of the whole brain and cerebellum was performed in Amira 5.5.0 (Thermo Fisher Scientific, USA). Ancestral cerebellar volumes (relative to whole brain) were obtained using a maximum-likelihood estimator assuming the tree as unit edge lengths in the R package ape v5.6-2 (*72*) and visualized by mapping changes onto phylogeny using the R package phytools v1.0.3 (*73*). The effect of locomotion on cerebellar relative volume was assessed with phylogenetic ANOVA in the R package phytools v1.0.3 (*73*). Cerebellar neuronal densities from 18 squamate species were extracted from published data on the evolution of brain neuron numbers in amniotes (*47*).

## Supplementary Materials

### This PDF file includes:

Figs. S1 to S4 Tables S1 to S9 References
