# Supplementary material for: Reconstructing the origin and early evolution of the snake brain
Source: Sci Adv. 2023 Sep 27;9(39):eadi6888. doi: 10.1126/sciadv.adi6888 (PMC10530081; doi:10.1126/sciadv.adi6888)
Supplement: Supplementary file 1 — Figs. S1 to S4 Tables S1 to S9 References [file sciadv.adi6888_sm.pdf]

Science Advances
AAAS

Supplementary Materials for

Reconstructing the origin and early evolution of the snake brain

Simone Macrì *et al.*

Corresponding author: Nicolas Di-Poï, nicolas.di-poi@helsinki.fi

Sci. Adv. **9**, eadi6888 (2023)
DOI: 10.1126/sciadv.adi6888

This PDF file includes:

Figs. S1 to S4
Tables S1 to S9
References

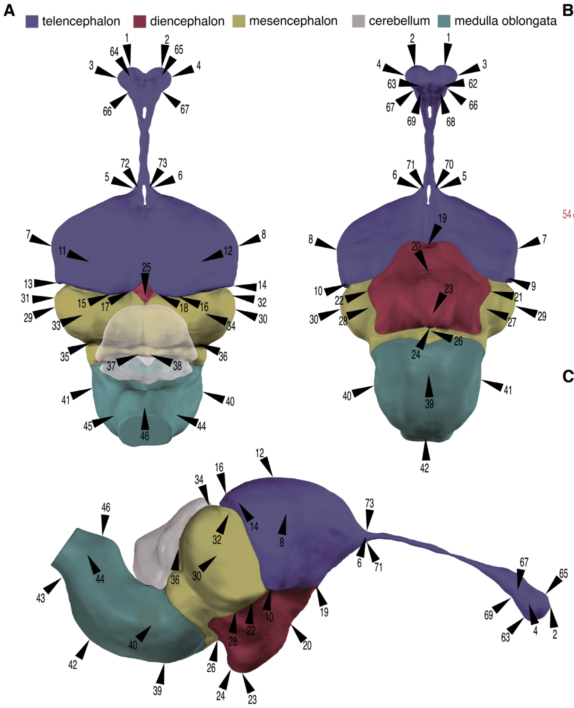

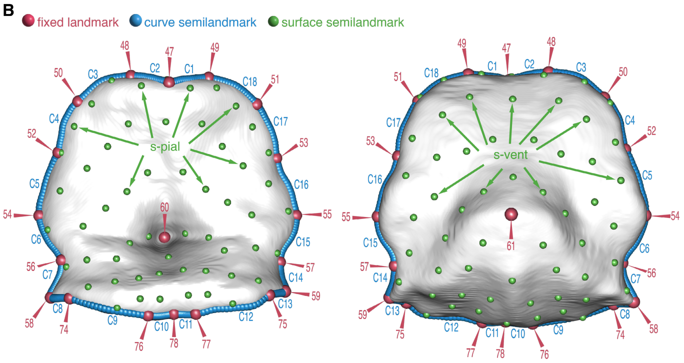

C

Landmark	3D model	Definition
1-61	whole brain	See our previous work (reference 41)
62-63[a]	whole brain	Ventral-most extent of the main olfactory bulb
64-65[a]	whole brain	Dorsal-most extent of the main olfactory bulb
66-67[a]	whole brain	Posterior limit of the main olfactory bulb along its lateral edge
68-69[a]	whole brain	Posterior limit of the main olfactory bulb along its ventral edge
70-71[a]	whole brain	Anteroventral margin of the cerebral hemisphere
72-73[a]	whole brain	Anterodorsal margin of the cerebral hemisphere
47-61	isolated cerebellum	See our previous work (reference 41)
74-78	isolated cerebellum	62-66 in our previous work (reference 41)
C1-C18[a]	isolated cerebellum	Curve semilandmarks (20 each curve) between two consecutive fixed landmarks along the cerbellar edge
s-pial[a]	isolated cerebellum	56 surface semilandmarks on the pial surface
s-vent[a]	isolated cerebellum	57 surface semilandmarks on the ventricular surface

[a]Additional landmark digitized in the present study

Fig. S1. Landmark and semilandmark data. (**A**) Position of 3D fixed anatomical landmarks on the whole brain of the lizard *Agama agama* in dorsal (top left panel), ventral (top right), and lateral (bottom) views. Major brain subdivisions are shown in different colors. (**B**) Position of 3D fixed anatomical landmarks and of semilandmarks on curves and surface on the pial (left) and ventricular (right) surface of *Agama agama* isolated cerebellum. (**C**) Table showing the definition of 3D fixed landmarks and semilandmarks with associated number (see **A**, **B**) and 3D model.

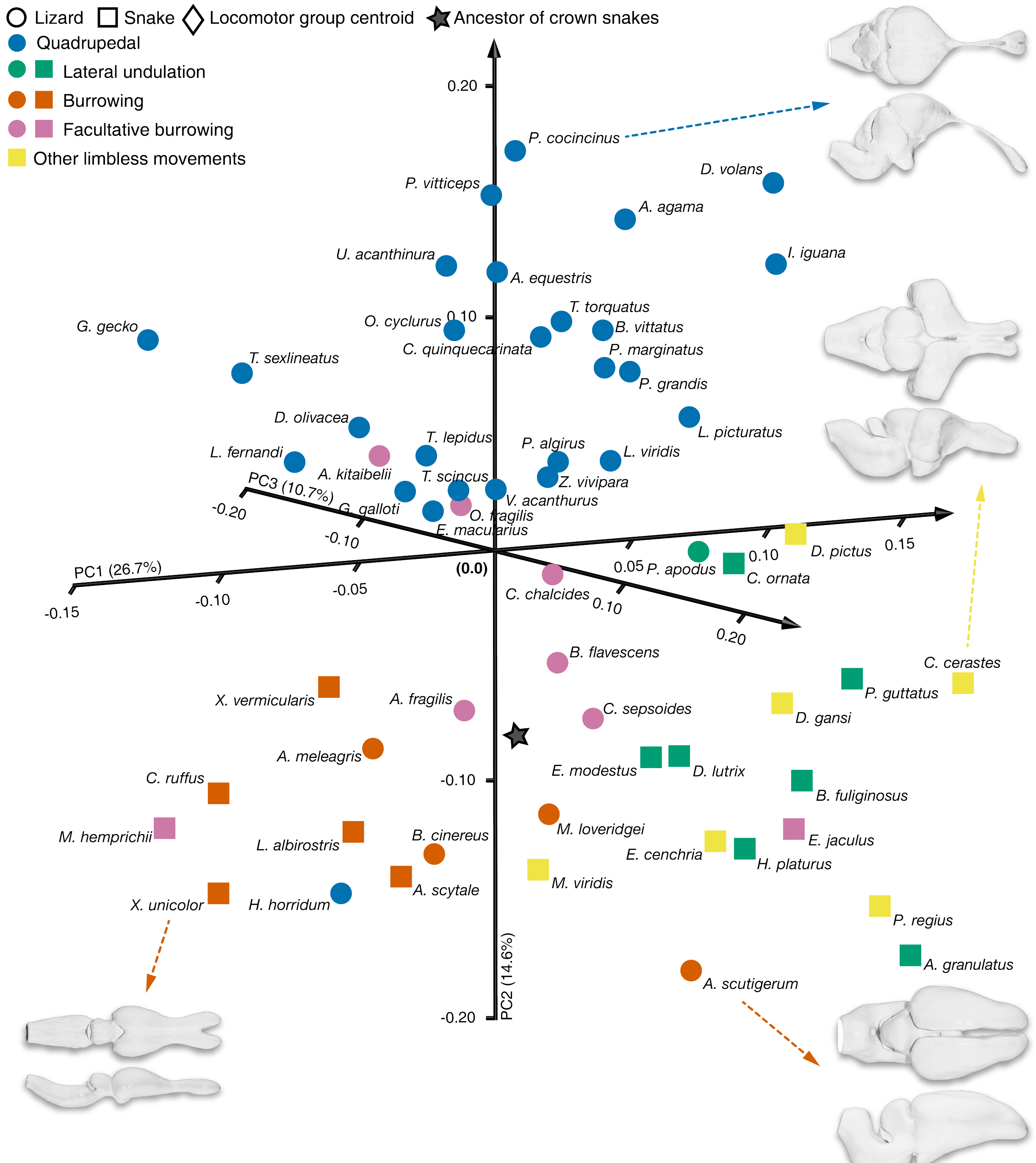

Fig. S2. Morphospace of the whole brain with species names. 3D morphospace (with species names indicated) representing whole-brain shape distribution of extant snakes (colored squares) and lizards (colored circles) with different locomotor modes (see color code and symbols in top left corner). The estimated position of the ancestor of crown snakes is marked by a black star. Whole-brain 3D models, indicated by colored dashed arrows and relative to the species located at the extremes of the first two principal component (PC) axes, are shown in dorsal (top) and lateral views (bottom). Numbers in brackets show the percentage of variance explained by each of the main PC axes.

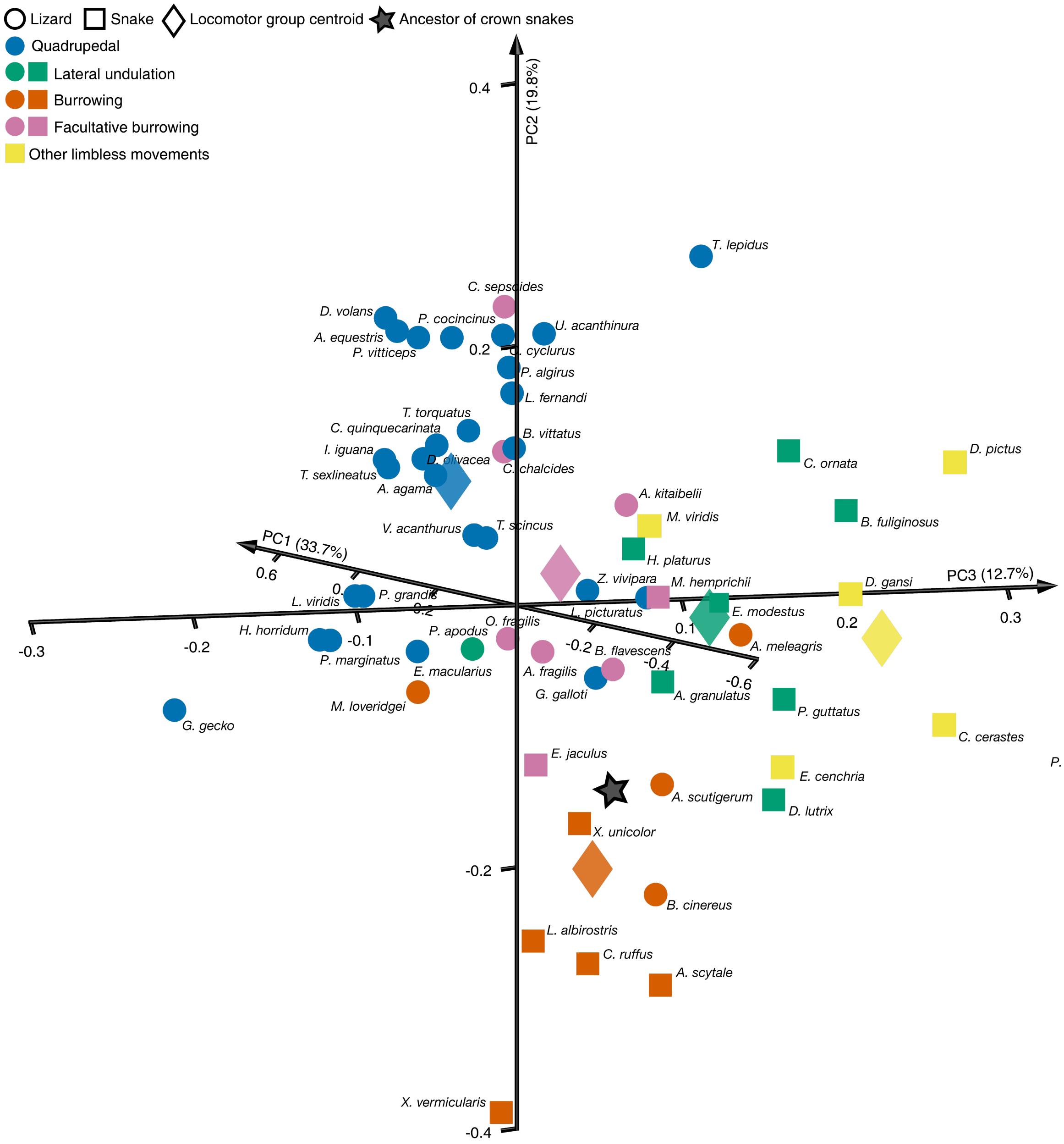

Fig. S3. Morphospace of the cerebellum obtained with a 15 fixed-landmark configuration. 3D morphospace (with species names indicated) showing the distribution of the cerebellar shape, defined by 15 fixed landmarks, of snakes (colored squares) and lizards (colored circles) with different locomotor modes (see color code in top left corner). The estimated position of the ancestor of crown snakes is marked by a black star. Numbers in brackets indicate the percentage of variance explained by each of the main PC axes, and centroids are shown for each locomotor group (colored rhombus).

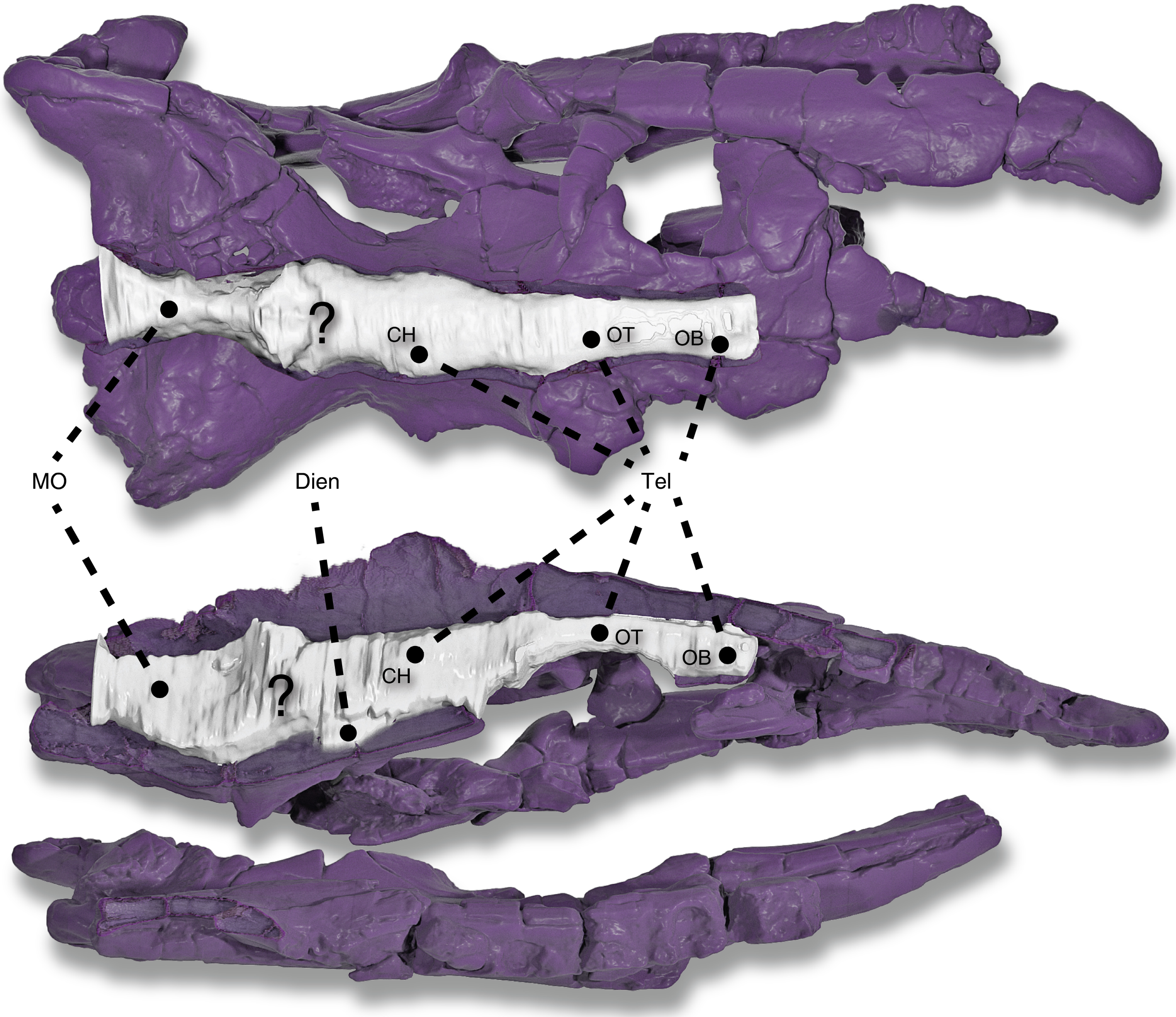

Fig. S4. Morphology and spatial relationships of the skull and the endocast of *Dinilysia patagonica*. 3D rendering of the skull and reconstructed endocast of the fossil *D. patagonica*, highlighting their spatial configuration and the limited potential of the endocast to resolve the morphological features of midbrain and hindbrain structures, including the cerebellum. Anatomical abbreviations: CH, cerebral hemisphere; Dien, diencephalon; MO, medulla oblongata; OB, olfactory bulb; OT, olfactory tract; Tel, telencephalon.

Group	Species	Family	Anatomical and energy efficiency features	Habitat preference	Movement types	Locomotor modes	References	Specimen source[a]	Whole-brain volume (mm^3)	Cerebellum volume (mm^3)
Lizard	*Ablepharus kitaibelii*	Scincidae	Slightly reduced forelimbs, elongated body	Facultative burrower (leaf litter)	Slow lateral undulation	Facultative burrowing	41	Previous work (reference 1)	9.267	1.532
Lizard	*Acontias meleagris*	Scincidae	Limbless, elongated body, cylindrical skull	Burrower	Modified concertina or rectilinear	Burrowing	41	Previous work (reference 1)	22.051	0.286
Snake	*Acrochordus granulatus*	Acrochordidae	Limbless	Aquatic	Lateral undulation	Lateral undulation	74	Morphosource UF:Herp:87078	32.839	1.992
Lizard	*Agama agama*	Agamidae	Intact limbs	Terrestrial	Quadrupedal terrestrial	Quadrupedal	41	Previous work (reference 1)	121.545	5.951
Lizard	*Amphisbaena scutigerum*	Amphisbaenidae	Limbless, elongated body, cylindrical skull, short tail	Burrower	Modified concertina or rectilinear	Burrowing	41	Previous work (reference 1)	33.503	0.445
Lizard	*Anguis fragilis*	Anguidae	Limbless, elongated body	Facultative burrower (leaf litter or terrestrial)	Slow lateral undulation	Facultative burrowing	41	Previous work (reference 1)	38.384	1.185
Snake	*Anilius scytale*	Aniliidae	Limbless (vestigial hindlimb), cylindrical skull, short tail, smooth scales	Burrower	Modified concertina or rectilinear	Burrowing	17,75-77	Morphosource ummz:herps:248356	24.679	0.373
Lizard	*Anolis equestris*	Dactyloidae	Intact limbs	Arboreal	Quadrupedal arboreal	Quadrupedal	78,79	FS, this work	84.113	3.41
Lizard	*Bachia flavescens*	Scincidae	Strongly reduced limbs, elongated body	Facultative burrower (leaf litter)	Slow lateral undulation	Facultative burrowing	41	Previous work (reference 1)	21.817	1.068
Lizard	*Basiliscus vittatus*	Corytophanidae	Intact limbs	Aerial/Semi-arboreal	Quadrupedal arboreal with facultative bipedal	Quadrupedal	41	Previous work (reference 1)	186.796	3.716
Lizard	*Blanus cinereus*	Blanidae	Limbless, elongated body, cylindrical skull, short tail, smooth scales	Burrower	Modified concertina or rectilinear	Burrowing	41	Previous work (reference 1)	8.081	0.161
Snake	*Boaedon fuliginosus*	Lamprophiidae	Limbless	Terrestrial	Lateral undulation	Lateral undulation	41	Previous work (reference 1)	43.737	1.203
Snake	*Cerastes cerastes*	Viperidae	Limbless	Terrestrial	Sidewinding	Other limbless movements	41	Previous work (reference 1)	48.253	1.532
Lizard	*Chalcides chalcides*	Scincidae	Strongly reduced limbs, elongated body	Facultative burrower (leaf litter)	Slow lateral undulation	Facultative burrowing	41	Previous work (reference 1)	34.635	1.328
Lizard	*Chalcides sepsoides*	Scincidae	Strongly reduced forelimbs, elongated body	Facultative burrower (sand-swimmer)	Slow lateral undulation	Facultative burrowing	41	Previous work (reference 1)	14.833	0.91
Snake	*Chrysopelea ornata*	Colubridae	Limbless	Aerial/Arboreal	Lateral undulation	Lateral undulation	41	Previous work (reference 1)	108.561	2.092
Lizard	*Ctenosaura quinquecarinata*	Iguanidae	Intact limbs	Arboreal	Quadrupedal arboreal	Quadrupedal	80	FS, this work	124.928	3.753

Snake	*Cylindrophis ruffus*	Cylindrophiidae	Limbless (vestigial hindlimb), smooth scales, no neck constriction, very short tail	Burrower	Modified concertina or rectilinear	Burrowing	17,77,81,82	Morphosource UF:Herp:143722	76.511	0.842
Lizard	*Dasia olivacea*	Scincidae	Intact limbs	Arboreal	Quadrupedal arboreal	Quadrupedal	41	Previous work (reference 1)	82.01	1.573
Snake	*Dasypeltis gansi*	Colubridae	Limbless	Arboreal	Arboreal concertina	Other limbless movements	41	Previous work (reference 1)	33.552	1.764
Snake	*Dendrelaphis pictus*	Colubridae	Limbless	Arboreal	Arboreal concertina	Other limbless movements	41	Previous work (reference 1)	46.374	1.859
Snake	*Dinilysia patagonica*	Fossil	?	?	?	?	11,18	Morphosource macn:rn:1013	?	?
Lizard	*Draco volans*	Agamidae	Intact limbs	Aerial/Arboreal	Quadrupedal arboreal with facultative aerial	Quadrupedal	41	Previous work (reference 1)	156.683	3.861
Snake	*Duberria lutrix*	Pseudoxyrhophiidae	Limbless	Terrestrial	Lateral undulation	Lateral undulation	83	MS 1424	16.27	0.965
Snake	*Eirenis modestus*	Colubridae	Limbless	Terrestrial	Lateral undulation	Lateral undulation	84	MS 1588	16.19	1.557
Snake	*Epicrates cenchria*	Boidae	Limbless (vestigial hindlimb)	Terrestrial	Rectilinear	Other limbless movements	41	Previous work (reference 1)	238.827	1.534
Snake	*Eryx jaculus*	Boidae	Limbless	Facultative burrower (sand-swimmer)	Slow lateral undulation	Facultative burrowing	41	Previous work (reference 1)	43.766	0.558
Lizard	*Eublepharis macularius*	Eublepharidae	Intact limbs	Terrestrial	Quadrupedal terrestrial	Quadrupedal	41	Previous work (reference 1)	70.951	4.185
Lizard	*Gallotia galloti*	Lacertidae	Intact limbs	Terrestrial	Quadrupedal terrestrial	Quadrupedal	85	MS 1984	135.685	2.091
Lizard	*Gekko gecko*	Gekkonidae	Intact limbs	Arboreal	Quadrupedal arboreal	Quadrupedal	41	Previous work (reference 1)	156.904	3.267
Lizard	*Heloderma horridum*	Helodermatidae	Intact limbs	Semi-arboreal	Quadrupedal arboreal	Quadrupedal	86	Morphosource UF:Herp:42033	501.527	1.725
Snake	*Hydrophis platurus*	Elapidae	Limbless	Aquatic	Lateral undulation	Lateral undulation	41	Previous work (reference 1)	35.174	2.334
Lizard	*Iguana iguana*	Iguanidae	Intact limbs	Arboreal	Quadrupedal arboreal	Quadrupedal	17,78,87	FS, this work	194.083	3.942
Lizard	*Lacerta viridis*	Lacertidae	Intact limbs	Terrestrial	Quadrupedal terrestrial	Quadrupedal	17,88,89	MS 2455	53.849	2.173
Lizard	*Lepidothyris fernandi*	Scincidae	Intact limbs	Terrestrial	Quadrupedal terrestrial	Quadrupedal	41	Previous work (reference 1)	128.605	2.342
Snake	*Liotyphlops albirostris*	Anomalepididae	Limbless (vestigial hindlimb), cylindrical skull, short tail, smooth scales	Burrower	Modified concertina or rectilinear	Burrowing	17,75,77	Morphosource uv:cpz-uv:7289	1447.887	0.203
Lizard	*Lygodactylus picturatus*	Gekkonidae	Intact limbs	Arboreal	Quadrupedal arboreal	Quadrupedal	41	Previous work (reference 1)	15.993	2.176
Lizard	*Melanoseps loveridgei*	Scincidae	Limbless, elongated body, cylindrical skull	Burrower	Modified concertina or rectilinear	Burrowing	41,90	Previous work (reference 1)	13.623	0.257
Snake	*Micrurus hemprichii*	Elapidae	Limbless	Facultative burrower (leaf litter)	Slow lateral undulation	Facultative burrowing	91,92	Morphosource ummz:herps:246857	46.935	0.609
Snake	*Morelia viridis*	Boidae	Limbless (vestigial hindlimb)	Arboreal	Arboreal concertina	Other limbless movements	93	Morphosource UF:Herp:120539	179.505	2.164

Snake	*Najash rionegrina*	Fossil	Limbless (vestigial hindlimb)	?	?	?	12,19	Reference 73	?	?
Lizard	*Ophiodes fragilis*	Anguidae	Limbless (vestigial hindlimb), elongated body	Facultative burrower (leaf litter or terrestrial)	Slow lateral undulation	Facultative burrowing	41	Previous work (reference 1)	45.687	0.876
Lizard	*Oplurus cyclurus*	Opluridae	Intact limbs	Arboreal	Quadrupedal arboreal	Quadrupedal	17,94	FS, this work	183.7	5.085
Snake	*Pantherophis guttatus*	Colubridae	Limbless	Terrestrial	Lateral undulation	Lateral undulation	41	Previous work (reference 1)	59.859	1.437
Lizard	*Phelsuma grandis*	Gekkonidae	Intact limbs	Arboreal	Quadrupedal arboreal	Quadrupedal	41	Previous work (reference 1)	45.705	2.628
Lizard	*Physignathus cocincinus*	Agamidae	Intact limbs	Arboreal	Quadrupedal arboreal	Quadrupedal	17,95,96	FS, this work	187.227	5.006
Lizard	*Plestiodon marginatus*	Scincidae	Intact limbs	Terrestrial	Quadrupedal terrestrial	Quadrupedal	41	Previous work (reference 1)	12.612	2.371
Lizard	*Pogona vitticeps*	Agamidae	Intact limbs	Terrestrial	Quadrupedal terrestrial	Quadrupedal	41	Previous work (reference 1)	102.852	4.365
Lizard	*Psammodromus algirus*	Lacertidae	Intact limbs	Terrestrial	Quadrupedal terrestrial	Quadrupedal	17,97,98	MS 1198	43.793	1.898
Lizard	*Pseudopus apodus*	Anguidae	Limbless, elongated body	Terrestrial	Lateral undulation	Lateral undulation	41	Previous work (reference 1)	71.72	1.249
Snake	*Python regius*	Pythonidae	Limbless (vestigial hindlimb)	Terrestrial	Rectilinear	Other limbless movements	41	Previous work (reference 1)	220.58	1.655
Lizard	*Takydromus sexlineatus*	Lacertidae	Intact limbs	Semi-arboreal	Quadrupedal arboreal	Quadrupedal	41	Previous work (reference 1)	18.257	3.062
Lizard	*Teratoscincus scincus*	Gekkonidae	Intact limbs	Terrestrial	Quadrupedal terrestrial	Quadrupedal	41	Previous work (reference 1)	21.609	2.999
Lizard	*Timon lepidus*	Lacertidae	Intact limbs	Terrestrial	Quadrupedal terrestrial	Quadrupedal	78	FS, this study	37.551	2.146
Lizard	*Tropidurus torquatus*	Tropiduridae	Intact limbs	Semi-arboreal	Quadrupedal arboreal with facultative bipedal	Quadrupedal	41	Previous work (reference 1)	76.583	3.595
Lizard	*Uromastyx acanthinura*	Agamidae	Intact limbs	Terrestrial	Quadrupedal terrestrial	Quadrupedal	99	MS 1860	370.033	4.893
Lizard	*Varanus acanthurus*	Varanidae	Intact limbs	Terrestrial	Quadrupedal terrestrial	Quadrupedal	17,100	FS, this study	235.319	3.457
Snake	*Xenopeltis unicolor*	Xenopeltidae	Limbless, smooth scales, flat head, short tail	Burrower	Modified concertina or rectilinear	Burrowing	17,101,102	Morphosource UF:herp:191595	238.955	0.639
Snake	*Xerotyphlops vermicularis*	Typhlopidae	Limbless (vestigial hindlimb), cylindrical skull, smooth scales	Burrower	Modified concertina or rectilinear	Burrowing	41	Previous work (reference 1)	4.625	0.022
Lizard	*Zootoca vivipara*	Lacertidae	Intact limbs	Terrestrial	Quadrupedal terrestrial	Quadrupedal	17,103	MS 219	30.145	1.927

[a] MS, museum sample (Finnish Museum of Natural History, Helsinki, Finland); FS, freshly dissected sample

Table S1. List of the material analyzed, including parameters (anatomical criteria, habitat preference, and movement types) used to define the main locomotor categories for extant squamate species (see Methods and reference 1 for descriptions of categorization).

Partition	*K*	*P* value[a]
Whole brain	0.721	0.001
Cerebellum (15 landmarks)	0.623	0.001
Cerebellum (high density)	0.609	0.001
Telencephalon	0.710	0.001
Diencephalon	0.507	0.001
Mesencephalon	0.591	0.001
Medulla oblongata	0.445	0.007

[a] *P* values from permutation tests (10,000 permutation rounds).

Table S2. Estimation of phylogenetic signal using a multivariate *K*-statistic for the whole brain and individual brain subdivisions.

Partition	Effect	df[a]	SS[b]	MS[c]	Rsq	F	Z	*P* value[d]
Whole brain	Locomotion	4	0.003049029	0.000762257	0.120252725	1.81114355	2.214601307	**0.012***
	Residuals	53	0.022306147	0.000420871	0.879747275			
	Total	57	0.025355176					
Cerebellum (15 landmarks)	Locomotion	4	0.006151354	0.001537839	0.13718049	2.10663005	2.530497759	**0.005****
	Residuals	53	0.038689965	0.000729999	0.86281951			
	Total	57	0.04484132					
Cerebellum (high density)	Locomotion	4	0.006569076	0.001642269	0.159456054	2.513601726	3.871279374	**0.0001****
	Residuals	53	0.034627703	0.000653353	0.840543946			
	Total	57	0.041196779					
Telencephalon	Locomotion	4	0.003489693	0.000872423	0.12013948	1.80920515	2.26598343	**0.01***
	Residuals	53	0.025557317	0.000482214	0.87986052			
	Total	57	0.02904701					
Diencephalon	Locomotion	4	0.004802136	0.001200534	0.14161466	2.18595801	2.673955107	**0.004****
	Residuals	53	0.029107744	0.000549203	0.85838534			
	Total	57	0.033909881					
Mesencephalon	Locomotion	4	0.002580401	0.0006451	0.07933385	1.14175315	0.620104983	0.269
	Residuals	53	0.029945454	0.000565009	0.92066615			
	Total	57	0.032525855					
Medulla oblongata	Locomotion	4	0.002098242	0.000524561	0.12151996	1.83286972	1.715437527	**0.042***
	Residuals	53	0.015168403	0.000286196	0.87848004			
	Total	57	0.017266645					

[a] df, degree of freedom.
[b] SS, sum of squares.
[c] MS, mean squares.
[d] Significant (**P* values < 0.05) and highly significant (***P* values < 0.01) values from permutation tests (10,000 permutation rounds) are bolded.

Table S3. Phylogenetic ANOVA assessing the effect of locomotion on whole-brain and each brain subdivision morphology.

Whole brain

Locomotor mode[a]	d[b]	UCL (95%)[c]	Z	Pr > d[d]
Fbu *Vs* Bu	0.010191291	0.02002268	-0.06887909	0.519
Fbu *Vs* Lu	0.017426445	0.02115159	1.24260212	0.122
Fbu *Vs* Olm	0.025641722	0.02273893	1.92186579	**0.024**
Fbu *Vs* Qu	0.009308447	0.01645694	0.20284755	0.424
Bu *Vs* Lu	0.0166446	0.02033282	1.2175348	0.127
Bu *Vs* Olm	0.024107971	0.0218779	1.87485099	**0.028**
Bu *Vs* Qu	0.011371715	0.01580803	0.87349621	0.211
Lu *Vs* Olm	0.011797276	0.0226722	0.05387478	0.471
Lu *Vs* Qu	0.010187238	0.01693507	0.44146136	0.338
Olm *Vs* Qu	0.017995595	0.01836714	1.56586126	**0.055**

Cerebellum (15 landmarks)

Locomotor mode[a]	d[b]	UCL (95%)[c]	Z	Pr > d[d]
Fbu *Vs* Bu	0.02673049	0.02435855	2.0025991	**0.023**
Fbu *Vs* Lu	0.02113006	0.02525288	0.9809355	0.166
Fbu *Vs* Olm	0.0347512	0.02743435	2.5682033	**0.003**
Fbu *Vs* Qu	0.01800893	0.02019475	1.225045	0.114
Bu *Vs* Lu	0.02138522	0.02437234	1.1548745	0.127
Bu *Vs* Olm	0.03118153	0.0264356	2.2587048	**0.011**
Bu *Vs* Qu	0.02303036	0.01922534	2.3438998	**0.008**
Lu *Vs* Olm	0.02014017	0.02736442	0.5593464	0.288
Lu *Vs* Qu	0.01063694	0.02032234	-0.6459955	0.737
Olm *Vs* Qu	0.02446152	0.02252523	1.9354736	**0.026**

Cerebellum (high density)

Locomotor mode[a]	d[b]	UCL (95%)[c]	Z	Pr > d[d]
Fbu *Vs* Bu	0.02195924	0.0161132	3.0397186	**0.0008**
Fbu *Vs* Lu	0.02032041	0.0217277	1.1519394	0.135
Fbu *Vs* Olm	0.0285303	0.02856012	1.7033462	0.052
Fbu *Vs* Qu	0.0137953	0.01607667	0.8428407	0.206
Bu *Vs* Lu	0.02336988	0.01912008	2.5927691	**0.003**
Bu *Vs* Olm	0.03000432	0.02562144	2.5974583	**0.002**
Bu *Vs* Qu	0.02250359	0.01571961	3.1685062	**0.0007**
Lu *Vs* Olm	0.01387462	0.01914582	-0.116553	0.546
Lu *Vs* Qu	0.01091382	0.01628172	-0.9220724	0.814
Olm *Vs* Qu	0.02016992	0.02144168	1.1076237	0.147

[a] Locomotor mode: Burrowing (Bu); Facultative burrowing (Fbu); Lateral undulation (Lu); Other limbless movements (Olm); Quadrupedal (Qu).
[b] Distance between LS means.
[c] Upper confidence limits from the distributions of pairwise distances.
[d] Significant *P* values between pairwise means (< 0.05) from permutation tests (10,000 permutation rounds) are bolded.

Table S4. Pairwise differences of locomotor modes from phylogenetic ANOVA for the whole-brain and cerebellum configurations.

Observation	Predicted locomotor group[a]	Whole brain[b] (6 PCs) Accuracy = 0.626; k = 0.453	Cerebellum[b] (15 landmarks,5 PCs) Accuracy = 0.655; k = 0.502	Cerebellum[b] (high density,7 PCs) Accuracy = 0.626; k = 0.468	Diencephalon[b] (4 PCs) Accuracy = 0.513; k = 0.287
Ancestor of crown snakes	Bu	0.292	**0.671**	**0.938**	0.325
	Fbu	0.337	0.051	0.031	0.136
	Lu	0.0009	0.248	0.026	0.112
	Olm	0.0001	0.025	0.0006	0.061
	Qu	**0.37**	0.005	0.0044	**0.366**

[a] Burrowing (Bu); Facultative burrowing (Fbu); Lateral undulation (Lu); Other limbless movements (Olm); Quadrupedal (Qu).
[b] The locomotor mode with the highest predicted probability is bolded in each subdivision analyzed.

Table S5. Locomotor mode predictions for the ancestor of crown snakes based on whole-brain and relevant major brain subdivision morphology.

Whole brain (6 PCs)

Observation	Predicted locomotor group[a]	Probability[b]
Ancestor of crown snakes	Bu	0.694
	Fbu	**0.763**
	Lu	0.018
	Olm	0.004
	Qu	0.470

Cerebellum (high density, 7 PCs)

Observation	Predicted locomotor group[a]	Probability[b]
Ancestor of crown snakes	Bu	**0.962**
	Fbu	0.264
	Lu	0.396
	Olm	0.065
	Qu	0.040

[a] Burrowing (Bu); Facultative burrowing (Fbu); Lateral undulation (Lu); Other limbless movements (Olm); Quadrupedal (Qu).
[b] The locomotor mode with the highest predicted probability is bolded.

Table S6. Locomotor mode typicality probabilities for the ancestor of crown snakes based on whole-brain and cerebellar morphology.

Locomotor mode[a,b]	Bu[c]	Fbu[c]	Lu[c]	Olm[c]	Qu[c]
Bu[c]	-	0.167	**0.028**	**0.014**	**0.0001**
Fbu[c]	0.167	-	0.368	0.312	**0.0001**
Lu[c]	**0.028**	0.368	-	0.717	**0.032**
Olm[c]	**0.014**	0.312	0.717	-	0.063
Qu[c]	**0.0001**	**0.0001**	**0.032**	0.063	-

[a] *P* values are indicated in upper and lower off-diagonal cells.
[b] Significant *P* values (< 0.05) from permutation tests (10,000 permutation rounds) are bolded.
[c] Burrowing (Bu); Facultative burrowing (Fbu); Lateral undulation (Lu); Other limbless movements (Olm); Quadrupedal (Qu).

Table S7. Pairwise differences of locomotor modes from phylogenetic ANOVA for the relative cerebellum volume.

Observation	Predicted locomotor group[a]	Relative cerebellar volume[b] Accuracy = 0.665 k = 0.519
Ancestor of crown snakes	Bu	**0.342**
	Fbu	0.318
	Lu	0.199
	Olm	0.124
	Qu	0.015

[a] Burrowing (Bu); Facultative burrowing (Fbu); Lateral undulation (Lu); Other limbless movements (Olm); Quadrupedal (Qu).
[b] The locomotor mode with the highest predicted probability is bolded.

Table S8. Locomotor mode predictions for the ancestor of crown snakes based on relative cerebellar volume.

Observation	Predicted locomotor group[a]	Probability[b]
Ancestor of crown snakes	Bu	0.663
	Fbu	**0.747**
	Lu	0.309
	Olm	0.235
	Qu	0.003

[a] Burrowing (Bu); Facultative burrowing (Fbu); Lateral undulation (Lu); Other limbless movements (Olm); Quadrupedal (Qu).
[b] The locomotor mode with the highest predicted probability is bolded.

Table S9. Locomotor mode typicality probabilities for the ancestor of crown snakes based on relative cerebellar volume.

REFERENCES AND NOTES

1. M. W. Caldwell, *The Origin of Snakes: Morphology and the Fossil Record* (CRC Press, 2019).

2. D. J. Gower, H. Zaher, *The Origin and Early Evolutionary History of Snakes* (Cambridge Univ. Press, 2022).

3. M. W. Caldwell, M. S. Y. Lee, A snake with legs from the marine Cretaceous of the Middle East. *Nature* **386**, 705–709 (1997).

4. M. S. Y. Lee, G. L. Bell Jr., M. W. Caldwell, The origin of snake feeding. *Nature* **400**, 655–659 (1999).

5. M. S. Y. Lee, Molecular evidence and marine snake origins. *Biol. Lett.* **1**, 227–230 (2005).

6. C. I. Caprette, M. S. Y. Lee, R. Shine, A. Mokany, J. F. Downhover, The origin of snakes (Serpentes) as seen through eye anatomy. *Biol. J. Linn. Soc.* **81**, 469–482 (2004).

7. J. D. Scanlon, M. S. Lee, The Pleistocene serpent Wonambi and the early evolution of snakes. *Nature* **403**, 416–420 (2000).

8. M. S. Y. Lee, J. D. Scanlon, Snake phylogeny based on osteology, soft anatomy and ecology. *Biol. Rev. Camb. Philos. Soc.* **77**, 333–401 (2002).

9. J. D. Scanlon, Skull of the large non-macrostomatan snake *Yurlunggur* from the Australian Oligo-Miocene. *Nature* **439**, 839–842 (2006).

10. A. Watanabe, A. C. Fabre, R. N. Felice, J. A. Maisano, J. Müller, A. Herrel, A. Goswami, Ecomorphological diversification in squamates from conserved pattern of cranial integration. *Proc. Natl. Acad. Sci. U.S.A.* **116**, 14688–14697 (2019).

11. L. N. Triviño, A. M. Albino, M. T. Dozo, J. D. Williams, First natural endocranial cast of a fossil snake (Cretaceous of Patagonia, Argentina). *Anat. Rec. (Hoboken)* **301**, 9–20 (2018).

12. S. Apesteguía, H. Zaher, A Cretaceous terrestrial snake with robust hindlimbs and a sacrum. *Nature* **440**, 1037–1040 (2006).

13. N. R. Longrich, B.-A. S. Bhullar, J. A. Gauthier, A transitional snake from the Late Cretaceous period of North America. *Nature* **488**, 205–208 (2012).

14. D. M. Martill, H. Tischlinger, N. R. Longrich, A four-legged snake from the Early Cretaceous of Gondwana. *Science* **349**, 416–419 (2015).

15. H. Yi, M. A. Norell, The burrowing origin of modern snakes. *Sci. Adv.* **1**, e1500743 (2015).

16. M. W. Caldwell, R. L. Nydam, A. Palci, S. Apesteguía, The oldest known snakes from the middle Jurassic-Lower Cretaceous provide insights on snake evolution. *Nat. Commun.* **6**, 5996 (2015).

17. F. O. Da Silva, A. C. Fabre, Y. Savriama, J. Ollonen, K. Mahlow, A. Herrel, J. Müller, N. Di-Poï, The ecological origins of snakes as revealed by skull evolution. *Nat. Commun.* **9**, 376 (2018).

18. H. Zaher, C. A. Scanferla, The skull of the Upper Cretaceous snake *Dinilysia patagonica* Smith-Woodward, 1901, and its phylogenetic position revisited. *Zool. J. Linn. Soc.* **164**, 194–238 (2012).

19. F. F. Garberoglio, S. Apesteguía, T. R. Simões, A. Palci, R. O. Gómez, R. L. Nydam, H. C. E. Larsson, M. S. Y. Lee, M. W. Caldwell, New skulls and skeletons of the Cretaceous legged snake *Najash*, and the evolution of the modern snake body plan. *Sci. Adv.* **5**, eaax5833 (2019).

20. J. A. Gauthier, M. Kearney, J. A. Maisano, O. Rieppel, A. D. B. Behlke, Assembling the squamate tree of life: Perspectives from the phenotype and the fossil record. *Bull. Peabody Mus. Nat. Hist.* **53**, 3–308 (2012).

21. T. W. Reeder, T. M. Townsend, D. G. Mulcahy, B. P. Noonan, P. L. Wood Jr., J. W. Sites Jr., J. J. Wiens, Integrated analyses resolve conflicts over squamate reptile phylogeny and reveal unexpected placements for fossil taxa. *PLOS ONE* **10**, e0118199 (2015).

22. A. Y. Hsiang, D. J. Field, T. H. Webster, A. D. Behlke, M. B. Davis, R. A. Racicot, J. A. Gauthier, The origin of snakes: Revealing the ecology, behavior, and evolutionary history of early snakes using genomics, phenomics, and the fossil record. *BMC Evol. Biol.* **15**, 87 (2015).

23. R. A. Pyron, Novel approaches for phylogenetic inference from morphological data and total-evidence dating in squamate reptiles (lizards, snakes, and amphisbaenians). *Syst. Biol.* **66**, 38–56 (2016).

24. T. R. Simões, M. W. Caldwell, M. Tałanda, M. Bernardi, A. Palci, O. Vernygora, F. Bernardini, L. Mancini, R. L. Nydam, The origin of squamates revealed by a Middle Triassic lizard from the Italian Alps. *Nature* **557**, 706–709 (2018).

25. A. Scanferla, Postnatal ontogeny and the evolution of macrostomy in snakes. *R. Soc. Open Sci.* **3**, 160612 (2016).

26. M. Bars-Closel, T. Kohlsdorf, D. S. Moen, J. J. Wiens, Diversification rates are more strongly related to microhabitat than climate in squamate reptiles (lizards and snakes). *Evolution* **71**, 2243–2261 (2017).

27. M. C. Grundler, D. L. Rabosky, Rapid increase in snake dietary diversity and complexity following the end-Cretaceous mass extinction. *PLOS Biol.* **19**, e3001414 (2021).

28. M. R. Moura, W. Jetz, Shortfalls and opportunities in terrestrial vertebrate species discovery. *Nat. Ecol. Evol.* **5**, 631–639 (2021).

29. A. Palci, M. N. Hutchinson, M. W. Caldwell, M. S. Y. Lee, The morphology of the inner ear of squamate reptiles and its bearing on the origin of snakes. *R. Soc. Open Sci.* **4**, 170685 (2017).

30. R. T. Figueroa, D. Goodvin, M. A. Kolmann, M. I. Coates, A. M. Caron, M. Friedman, S. Giles, Exceptional fossil preservation and evolution of the ray-finned fish brain. *Nature* **614**, 486–491 (2023).

31. A. M. Balanoff, G. S. Bever, "The role of endocasts in the study of brain evolution" in *Evolutionary neuroscience*, J. H. Kaas, Ed. (Academic Press, 2020), pp. 29–49.

32. T. B. Rowe, T. E. Macrini, Z. X. Luo, Fossil evidence on origin of the mammalian brain. *Science* **332**, 955–957 (2011).

33. A. M. Balanoff, G. S. Bever, T. B. Rowe, M. A. Norell, Evolutionary origins of the avian brain. *Nature* **501**, 93–96 (2013).

34. A. M. Clement, C. L. Mensforth, T. J. Challands, S. P. Collin, J. A. Long, Brain reconstruction across the fish-tetrapod transition; insights from modern amphibians. *Front. Ecol. Evol.* **9**, 640345 (2021).

35. A. Beaudet, The enigmatic origins of the human brain. *Science* **372**, 124–125 (2021).

36. D. G. Senn, R. G. Northcutt, The forebrain and midbrain of some squamates and their bearing on the origin of snakes. *J. Morphol.* **140**, 135–151 (1973).

37. J. A. Hopson, "Paleoneurology" in *Biology of Reptilia*, C. Gans, R. G. Northcutt, P. Ulinski, Eds. (Academy Press, 1979), vol. 9, pp. 39–139.

38. A. Scanferla, "A glimpse into the evolution of the ophidian brain" in *The Origin and Early Evolutionary History of Snakes*, D. J. Gower, H. Zaher, Eds. (Cambridge Univ. Press, 2022), pp. 294–315.

39. A. Paulina-Carabajal, P. Jiménez-Huidobro, L. N. Triviño, E. L. Stanley, H. Zaher, J. D. Daza, "A look in to the neurocranium of living and extinct Lepidosauria" in *Paleoneurology of Amniotes*, M. T. Dozo, A. Paulina-Carabajal, T. E. Macrini, E. Walsh, Eds. (Springer, 2023), pp. 123–177.

40. R. Allemand, R. Boistel, G. Daghfous, Z. Blanchet, R. Cornette, N. Bardet, P. Vincent, A. Houssaye, Comparative morphology of snake (Squamata) endocasts: Evidence of phylogenetic and ecological signals. *J. Anat.* **231**, 849–868 (2017).

41. S. Macrì, Y. Savriama, I. Khan, N. Di-Poï, Comparative analysis of squamate brains unveils multi-level variation in cerebellar architecture associated with locomotor specialization. *Nat. Commun.* **10**, 5560 (2019).

42. M. Segall, R. Cornette, A. R. Rasmussen, C. J. Raxworthy, Inside the head of snakes: Influence of size, phylogeny, and sensory ecology on endocranium morphology. *Brain Struct. Funct.* **226**, 2401–2415 (2021).

43. R. Allemand, J. Abdul-Sater, S. Macrì, N. Di-Poï, G. Daghfous, M. T. Silcox, Endocast, brain and bones: Correspondences and spatial relationships in squamates. *Anat. Rec.* 10.1002/ar.25142 (2023).

44. J. F. R. Tonini, K. H. Beard, R. B. Ferreira, W. Jetz, R. A. Pyron, Fully-sampled phylogenies of squamates reveal evolutionary patterns in threat status. *Biol. Conserv.* **204**, 23–31 (2016).

45. O. Rieppel, "Miniaturization in tetrapods: Consequences for skull morphology" in *Miniature Vertebrates: The Implications of Small Body Size*, P. J. Miller, Ed. (Clarendon Press, 1996), pp. 47–61.

46. T. S. Fachini, S. Onary, A. Palci, M. S. Y. Lee, M. Bronzati, A. S. Hsiou, Cretaceous blind snake from Brazil fills major gap in snake evolution. *iScience* **23**, 101834 (2020).

47. K. Kverková, L. Marhounová, A. Polonyiová, M. Kocourek, Y. Zhang, S. Olkowicz, B. Straková, Z. Pavelková, R. Vodička, D. Frynta, P. Němec, The evolution of brain neuron numbers in amniotes. *Proc. Natl. Acad. Sci. U.S.A.* **119**, e2121624119 (2022).

48. A. Feldman, N. Sabath, R. A. Pyron, I. Mayrose, S. Meiri, Body sizes and diversification rates of lizards, snakes, amphisbaenians and the tuatara. *Glob. Ecol. Biogeogr.* **25**, 187–197 (2016).

49. H. W. Greene, *Snakes: The Evolution of Mystery in Nature* (University of California Press, 1997).

50. C. Bardua, M. Wilkinson, D. J. Gower, E. Sherratt, A. Goswami, Morphological evolution and modularity of the caecilian skull. *BMC Evol. Biol.* **19**, 30 (2019).

51. A. Goswami, A. Watanabe, R. N. Felice, C. Bardua, A.-C. Fabre, P. D. Polly, High-density morphometric analysis of shape and integration: The good, the bad, and the not-really-a-problem. *Integr. Comp. Biol.* **59**, 669–683 (2019).

52. P. Gunz, P. Mitteroecker, F. L. Bookstein, "Semilandmarks in three dimensions" in *Modern Morphometrics in Physical Anthropology*, D. E. Slice, Ed. (Kluwer Academic/Plenum Publishers, 2005), pp. 73–98.

53. P. Gunz, P. Mitteroecker, Semilandmarks: A method for quantifying curves and surfaces. *Hystrix* **24**, 103–109 (2013).

54. S. Rolfe, S. Pieper, A. Porto, K. Diamond, J. Winchester, S. Shan, H. Kirveslahti, D. Boyer, A. Summers, A. M. Maga, SlicerMorph: An open and extensible platform to retrieve, visualize and analyse 3D morphology. *Methods Ecol. Evol.* **12**, 1816–1825 (2021).

55. R. Kikinis, S. D. Pieper, K. G. Vosburgh, “3D Slicer: A platform for subject-specific image analysis, visualization, and clinical support” in *Intraoperative Imaging and Image-Guided Therapy*, F. Jolesz, Ed. (Springer, 2014), pp. 277–289.

56. S. Schlager, “Morpho and Rvcg - shape analysis in R” in *Statistical Shape and Deformation Analysis*, G. Zheng, S. Li, G. Szekely, Eds. (Academic Press, 2017), pp. 217–256.

57. R Core Team, *R: A Language and Environment for Statistical Computing* (R Foundation for Statistical Computing, 2021).

58. W. P. Maddison, Squared-change parsimony reconstructions of ancestral states for continuous-valued characters on a phylogenetic tree. *Syst. Zool.* **40**, 304–314 (1991).

59. F. J. Rohlf, “Geometric morphometrics and phylogeny” in *Morphology, Shape and Phylogeny*, N. MacLeod, P. L. Forey, Eds. (CRC Press, 2002), pp. 175–193.

60. D. C. Adams, A generalized K statistic for estimating phylogenetic signal from shape and other high-dimensional multivariate data. *Syst. Biol.* **63**, 685–697 (2014).

61. D. C. Adams, M. L. Collyer, Phylogenetic ANOVA: Group-clade aggregation, biological challenges, and a refined permutation procedure. *Evolution* **72**, 1204–1215 (2018).

62. D. C. Adams, E. Otárola-Castillo, Geomorph: An r package for the collection and analysis of geometric morphometric shape data. *Methods Ecol. Evol.* **4**, 393–399 (2013).

63. W. N. Venables, B. D. Ripley, *Modern Applied Statistics with S.* (Springer, ed. 4, 2002).

64. M. Kuhn, Building predictive models inRUsing the caret Package. *J. Stat. Softw.* **28**, 1–26. (2008).

65. G. H. Albrecht, Assessing the affinities of fossils using canonical variates and generalized distances. *Hum. Evol.* **7**, 49–69 (1992).

66. S. R. Wilson, On comparing fossil specimens with population samples. *J. Hum. Evol.* **10**, 207–214 (1981).

67. R. H. MacArthur, On the relative abundance of bird species. *Proc. Natl. Acad. Sci. U.S.A.* **43**, 293–295 (1957).

68. M. Wang, S. M. Kornblau, K. R. Coombes, Decomposing the apoptosis pathway into biologically interpretable principal components. *Cancer Inform.* **17**, 1176935118771082 (2018).

69. F. L. Bookstein, Principal warps: Thin-plate splines and the decomposition of deformations. *IEEE Trans. Pattern Anal. Mach. Intell.* **11**, 567–585 (1989).

70. P. Antonio, B. Constantino, C. Silvia, M. Marina, P. Paolo, V. Alessio, R. Pasquale, Arothron: An R package for geometric morphometric methods and virtual anthropology applications. *Am. J. Phys. Anthropol.*, **176**, 144–151 (2021).

71. S. Garnier, N. Ross, R. Rudis, A. P. Camargo, M. Sciaini, C. Scherer, *viridis (Lite) - Colorblind-friendly color maps for R* (2021).

72. E. Paradis, K. Schliep, ape 5.0: An environment for modern phylogenetics and evolutionary analyses in R. *Bioinformatics* **35**, 526–528 (2019).

73. L. J. Revell, phytools: An R package for phylogenetic comparative biology (and other things). *Methods Ecol. Evol.* **3**, 217–223 (2012).

74. A. M. Lawing, J. J. Head, P. D. Polly, "The ecology of morphology: the ecometrics of locomotion and macroenvironment in North American snakes" in *Paleontology in Ecology and Conservation*, L. Louys, Ed. (Springer-Verlag, 2012), pp. 117–146.

75. A. Herrel, A. Lowie, A. Miralles, P. Gaucher, N. J. Kley, J. Measey and K. A. Tolley. Burrowing in blindsnakes: A preliminary analysis of burrowing forces and consequences for the evolution of morphology. *Anat. Rec.* **304**, 2292–2302 (2021).

76. G. F. Maschio, A. L. Prudente, F. Rodrigues, M. S. Hoogmoed, Food habits of Anilius scytale (Serpentes: Aniliidae) in the brazilian Amazonia. *Fortschr. Zool.* **27**, 184–190 (2010).

77. D. J. Gower, Scale microornamentation of uropeltid snakes. *J. Morphol.* **258**, 249–268 (2003).

78. A. Lowie, A. Herrel, V. Abdala, A. S. Manzano, A. C. Fabre, Does the morphology of the forelimb flexor muscles differ between lizards using different habitats? *Anat. Rec.* **301**, 424–433 (2018).

79. A. Schwartz, R. W. Henderson, *Amphibians and Reptiles of the West Indies: Descriptions, Distributions, and Natural History* (University of Florida Press, 1991).

80. A. C. Alberts, R. L. Carter, W. K. Hayes, E. P Martins, *Iguanas: Biology and Conservation* (University of California Press, 2005).

81. W. Himstedt, D. Gower, A. Kupfer, Field observations on the predation of the caecilian amphibian, genus Ichthyophis (Fitzinger, 1826), by the red-tailed pipe snake Cylindrophis ruffus (Laurenti, 1768). *Amphib.-reptil.* **24**, 212–215 (2003).

82. R. T. Hoser, Divisions within the snake genera *Cylindrophis* Wagler, 1828 (Cylindrophiidae Fitzinger, 1843) and *Anomochilus* Berg, 1901 (Anomochilidae Cundall, Wallach and Rossman, 1993). *Aust. J. Herpetol.* **16**, 31–38 (2013).

83. S. Spawls, K. Howell, R. C. Drewes, J. Ashe, *A Field Guide to the Reptiles of East Africa* (Princeton Academic Press, 2002).

84. E. N. Arnold, D. W. Ovenden, *Field Guide: Reptiles & Amphibians of Britain & Europe* (HarperCollins Publishers, 2002).

85. E. N. Arnold, J. A. Burton, D. W. Ovenden, *A Field Guide to the Reptiles and Amphibians of Britain and Europe* (Collins, 1978).

86. D. D. Beck, C. H. Lowe, Ecology of the beaded lizard, Heloderma horridum, in a tropical dry forest in Jalisco, Mexico. *J. Herpetol.* **25**, 395–406 (1991).

87. T. C. S. Avila-Pires, Lizards of brazilian Amazonia (Reptilia:Squamata). *Zool. Verh.* **299**, 1–706 (1995).

88. A. Pačuta, A. Žagar, B. Kočíková, V. Majláthová, A. D. Mihalca, I. Majláth, Time matters. Locomotor behavior of *Lacerta viridis* and *Lacerta agilis* in an open field maze. *Acta Ethol.* **21**, 91–99 (2018).

89. R. A. Avery, C. F. Mueller, S. M. Jones, J. A. Smith, D. J. Bond, Speeds and movement patterns of european lacertid lizards: A comparative study. *J. Herpetol.*, **21**, 324–329 (1987).

90. P. K. Malonza, B. A. Bwong, Life history notes on Loveridge'S Limbless Skink *Melanoseps Loveridgei* Brygoo & Roux-Estève, 1981 (Sauria: Scincidae: Feylininae). *Herpetotropicos* **5**, 115–117 (2011).

91. M. Martins, M. E. Oliveira, Natural history of snakes in forests of the Manaus region, central Amazonia, Brazil. *Herpetol. Nat. Hist.* **6**, 1–78 (1999).

92. J. A. Roze, *Coral Snakes of the Americas: Biology, Identification, and Venoms* (Krieger Publishing Company, 1996).

93. R. de Lang, G. Vogel, *The Snakes of Sulawesi: A Field Guide to the Land Snakes of Sulawesi with Identification Keys* (Edition Chimaira, 2005).

94. F. Glaw, M. Vences, *A Field Guide to the Amphibians and Reptiles of Madagascar* (Vences & Glaw, 1994).

95. R. C. Snyder, Quadrupedal and bipedal locomotion of lizards. *Copeia* **1952**, 64–70 (1952).

96. R. Meek, Thermoregulation and activity patterns in captive water dragons, Physignathus cocincinus, in a naturalistic environment. *Herpetol. J.* **9**, 137–146 (1999).

97. J. A. Díaz, L. M. Carrascal, Regional distribution of a mediterranean lizard: Influence of habitat cues and prey abundance. *J. Biogeogr.* **18**, 291–297 (1991).

98. D. Verwaijen, R. Van Damme, Foraging mode and its flexibility in lacertid lizards from Europe. *J. Herpetol.* **42**, 124–133 (2008).

99. H. H. Schleich, W. Kastle, K. Kabisch, *Amphibians and Reptiles of North Africa* (Koeltz, 1996).

100. R. L. Cieri, T. J. M. Dick, R. Irwin, D. Rumsey, C. J. Clemente, The scaling of ground reaction forces and duty factor in monitor lizards: Implications for locomotion in sprawling tetrapods. *Biol. Lett.* **17**, 20200612 (2021).

101. D. Escoriza, F. Amat, Habitat Partitioning and overlap by large lacertid lizards in southern Europe. *Diversity* **13**, 155 (2021)

102. O. S. G. Pauwels, P. David, P. F. A. Maderson, W. Dereck, C. Kumps, Dorsal scale microsculpture of *Xenopelis unicolor* (Serpentes, Xenopeltidae): Description and position among the ophidian microdermatoglyphic patterns, *Dumerilia* **4**, 99–111 (2000).

103. R. A. Avery, C. F. Mueller, J. A. Smith, D. J. Bond, The movement patterns of lacertid lizards: Speed, gait and pauses in *Lacerta vivipara*. *J. Zool.* **211**, 47–63 (1987).
